# Long‐Term Effects of Chronic Stress from Natural Hazards on Mental Health of Community‐Dwelling Filipino Elderly

**DOI:** 10.1002/alz70860_101917

**Published:** 2025-12-23

**Authors:** Krizelle Cleo Fowler, Jacqueline C Dominguez, Ma Fe P De Guzman, Clarissa del Moral

**Affiliations:** ^1^ Institute for Dementia Care Asia, Quezon City, Metro Manila, Philippines

## Abstract

**Background:**

The Philippines now ranked as the 1st most vulnerable to disaster risk and natural hazards among 173 countries in the World Risk Index 2024 study. In 2020 alone, during the COVID‐19 pandemic, the country was hit by 22 tropical cyclones. While mental health problems are being reported more by those living in disaster‐prone areas than from the general population, there is a paucity in studies exploring such more so on the community‐based elderly affected by frequent threat of natural disasters.

**Method:**

This study was conducted post‐COVID‐19 pandemic in Marikina City, Philippines, where a recent flooding also occurred during the November 2020 lockdown. The study had a mixed‐method cross‐sectional study design with participants senior citizens living in Marikina City (*n* = 627) during the time of data collection. This study explored using STORM‐32 as a tool to: (1) indicate flooding stress, and; (2) differentiate between cognitively normal and mildly impaired community‐based elderly. Other cognitive tests like MOCA‐P, MMSE, and AD8 were also administered and the results underwent consensus validation from psychologists, nurses, and psychiatrists.

Descriptive statistics and bivariate tests for associations and differences were used to explore the associations of chronic stress and mental health of the participants. Logistic regression models were also used for multivariate testing. For the qualitative data, thematic analysis was done.

**Result:**

There is high prevalence of cognitive impairment (65%) and psychiatric conditions (32.8%) in the community. Stressful experiences from natural hazards (STORM32 Total Score) have significant association with cognitive function and quality of life (MOCA‐P rho = 0.086; EQVAS rho = ‐0.103; *p* <0.05). Other than the measures for chronic stress, difficulty in meeting financial needs is a major stressor identified by participants and families during and post‐disaster.

**Conclusion:**

There is a high prevalence of cognitive and psychiatric conditions affecting this population. The study found an association, albeit weak, between chronic stress and cognitive or psychiatric diagnosis. Nonetheless, narratives from the community magnified the gaps on healthcare response to the elderly especially during emergencies. As a vulnerable population, improving care pathways specific to elderly needs during and post disaster events is of critical importance.

